# A kinematic and EMG dataset of online adjustment of reach-to-grasp movements to visual perturbations

**DOI:** 10.1038/s41597-021-01107-2

**Published:** 2022-01-21

**Authors:** Mariusz P. Furmanek, Madhur Mangalam, Mathew Yarossi, Kyle Lockwood, Eugene Tunik

**Affiliations:** 1 Department of Physical Therapy, Movement and Rehabilitation Sciences, Northeastern University, Boston, Massachusetts 02115 USA; 2 Institute of Sport Sciences, The Jerzy Kukuczka Academy of Physical Education in Katowice, 40-065 Katowice, Poland; 3 Department of Electrical and Computer Engineering, Northeastern University, Boston, Massachusetts 02115 USA; 4 Department of Bioengineering, Northeastern University, Boston, Massachusetts 02115 USA

**Keywords:** Motor control, Sensory processing

## Abstract

Control of reach-to-grasp movements for deft and robust interactions with objects requires rapid sensorimotor updating that enables online adjustments to changing external goals (e.g., perturbations or instability of objects we interact with). Rarely do we appreciate the remarkable coordination in reach-to-grasp, until control becomes impaired by neurological injuries such as stroke, neurodegenerative diseases, or even aging. Modeling online control of human reach-to-grasp movements is a challenging problem but fundamental to several domains, including behavioral and computational neuroscience, neurorehabilitation, neural prostheses, and robotics. Currently, there are no publicly available datasets that include online adjustment of reach-to-grasp movements to object perturbations. This work aims to advance modeling efforts of reach-to-grasp movements by making publicly available a large kinematic and EMG dataset of online adjustment of reach-to-grasp movements to instantaneous perturbations of object size and distance performed in immersive haptic-free virtual environment (hf-VE). The presented dataset is composed of a large number of perturbation types (10 for both object size and distance) applied at three different latencies after the start of the movement.

## Abstract (summary)

**Table:** 

Measurement(s)	kinematics • reach-to-grasp movements
Technology Type(s)	motion capture system • virtual reality • electromyography
Factor Type(s)	movement time [ms] • peak transport velocity [cm/s] • time to peak transport velocity [ms] • peak transport acceleration [cm/s2] • time to peak transport acceleration [ms] • peak transport deceleration [cm/s2] • time to peak transport deceleration [ms] • peak aperture [cm] • peak aperture velocity [cm/s] • time to peak aperture velocity [ms] • peak aperture deceleration [cm/s2] • time to peak aperture deceleration [ms] • opening time [ms] • closure time [ms] • opening distance [cm] • closure distance [cm] • transport velocity at CO [cm/s] • transport acceleration at CO [cm/s2] • peak closure velocity [cm/s] • peak closure deceleration [cm/s2]
Sample Characteristic - Organism	Homo sapiens

Machine-accessible metadata file describing the reported data: 10.6084/m9.figshare.16786258

## Background & Summary

Rarely do we appreciate the remarkable coordination involved in our routine reach-to-grasp movements, until control becomes impaired by neurological injuries such as stroke, neurodegenerative diseases, or even aging. Despite seemingly effortless execution, a simple reach-to-grasp movement involves complex, multi-level collective interaction of the brain, spinal cord, and the peripheral system which is tuned and coordinated through sensory experience. Efficient and flexible behavior in everyday context requires rapid online adjustments of reach-to-grasp to sudden changes in the position or affordances of the target object^1–10^. Accurate physical modeling of reach-to-grasp movement could advance applications in neurorehabilitation^11–13^, neural prostheses^14–16^, and robotics^17,18^. Critically, none of the numerous approaches to modeling of the coordination between reach and grasp components have been able to accurately replicate human behavior^19–21^. To advance our understanding of how reach-to-grasp movements are orchestrated and updated, the scientific community needs to turn to more sophisticated forms of characterizing this complex motor behavior. Large publicly available datasets of hand movements of grasping 3D objects^22–26^ recorded using video/Kinect/infrared motion capture have immensely benefited modeling efforts in grasp classification. However, these datasets are often collected with the explicit purpose of training robotic grasping and are not optimized for modeling of human manual behavior. Furthermore, there is no publicly available dataset that includes online adjustment of reach-to-grasp movements to perturbations, freely accessible to researchers from multiple fields for modeling and analytical means. In the absence of such a dataset, the existing models of reach-grasp coordination^19–21,27–32^—which typically rely on data collected under a limited set of task manipulations—have remained untested. A reach-to-grasp dataset that offers synchronized kinematic and electromyography (EMG) data for a broader set of conditions, including coordinated reach and grasp responses to perturbations of the task goal, would greatly aid future efforts directed toward modeling of reach-to-grasp movements.

The purpose of this report is to make publicly available rich dataset of kinematic and EMG collected during hand and arm movements as participants reached to grasp objects in an immersive haptic-free virtual environment (henceforth, hf-VE) that includes a large variety of object size and distance perturbations that required rapid online adjustments of movement to compensate for instantaneous perturbations of the goal. Moreover, in the interest of being able to study the extent to which there may be temporal dependencies related to the perturbation timing, the perturbations occurred at three different latencies after movement onset. This dataset has been collected using the state-of-the-art experimental setup developed over several years in the Movement Neuroscience Laboratory at Northeastern University, which includes the seamless integration of an immersive haptic-free virtual reality system, an active marker motion capture system, a wireless multichannel electromyography (EMG) recording system, and an immersive unity 3D-programmed hf-VE programmed in C# and Python. The hardware and software renderings were synchronized to obtain a ~13.3 ms feedback loop between participants’ hand movements and their virtual rendering corresponding to 75 Hz sampling of kinematic data (all kinematic data is provided after resampling to 100 Hz). The kinematic data primarily pertain to the transport and aperture aspects of reach-to-grasp movements. Whereas ‘transport’ refers to the motion of the hand towards the target object, aperture refers to the distance between the tips of the thumb and index finger that forms the enclosure around the target object. The dataset is organized as a Matlab (Mathworks Inc., Natick, MA) data structure (.mat) with kinematic and EMG data. The dataset’s novelty lies in a large number of conditions, including Perturbation Type (object size perturbation, object distance perturbation), Perturbation Timing (100 ms, 200 ms, and 300 ms after movement onset), and the combination of synchronized kinematic and EMG acquisition.

To our knowledge, no dataset exists for online adjustment of coordinated reach-to-grasp movements to perturbations of the task goal. Although some reach and grasp kinematics datasets (e.g., performing different reaches and grasps) are available, as well as forearm EMG datasets (e.g., performing hand gestures or freehand movements), they have several limitations for use in modeling online control of reach-to-grasp movements:

- **A focus on isolated grasp:** The available datasets focus solely on the grasp component with little to no mention of the reach component, or coordinated reach-to-grasp movements^33–39^.
- **Limited sample size:** Some of the existing datasets provide data only from a small number of participants (e.g., just one to four participants^35,40^, as opposed to a total of 20 participants in the present dataset, ten participants each for object size and distance perturbations), limiting their generalizability and the ability of modeling efforts to make generalizable inferential predictions.
- **No synchronization between kinematic and EMG data:** The available datasets offer either kinematics^38,39^ or EMG^41,42^ data but do not offer synchronized kinematic and EMG data.

This dataset overcomes the above-mentioned limitations. It is our hope that the data will be useful for modeling coordinated reach-to-grasp movements for both the basic and applied aspects of research. The present dataset consists of a Matlab/GNU Octave data structure (in.mat) with kinematic and EMG data (including maximal voluntary contraction or MVC for each muscle from which EMG was recorded). A separate.csv file contains sex, age, anthropometry data and laterality for all participants.

## Methods

### Participants and ethical requirements

Ten adults (eight men and two women; *mean* ± 1*s.d*. age: 22.5 ± 6.0 years, right-handed) participated in the size-perturbation study, and ten adults (eight men and two women; *mean* ± 1*s.d*. age: 25.3 ± 6.4 years, right-handed) participated in the distance-perturbation study. The participants were free of any muscular, orthopedic, or neurological health concerns. The participant pool comprised undergraduate and graduate students at Northeastern University. The participants were offered $10 per hour for participation. Each participant provided verbal and written consent approved by the Institutional Review Board (IRB) at Northeastern University. Some participants had previously participated in reach-to-grasp studies in our hf-VE, however, none of the participants reported extensive experience in virtual reality (e.g., gaming, simulations, etc.). To ensure adequate familiarization with the reach-to-grasp task in a virtual environment, all participants completed a training block of 120 reach-to-grasp trials [24 trials per object size: small (*w* × *h* × *d* = 3.5 × 8 × 2.5 cm), small-medium (4.5 × 8 × 2.5 cm), medium (5.5 × 8 × 2.5 cm), medium-large (6.5 × 8 × 2.5 cm), and large (7.5 × 8 × 2.5 cm) object placed at 30 cm; or 24 trials per object distance: medium object placed at near (20 cm), near-middle (25 cm), middle (30 cm), middle-far (35 cm), and far (40 cm) distances]. If participants felt comfortable after 60 trials, the training block was terminated, and the experimental trials began; otherwise, the participants completed all 120 training trials.

### Reach-to-grasp task, virtual environment, and kinematic/kinetic measurement

**Fig. 1:**
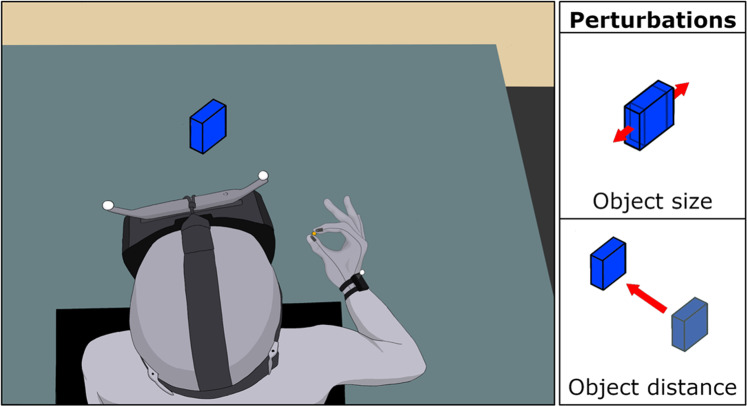
Using the pincer grip participants reached to grasp virtual objects of different sizes and placed at different distances from the initial position of their thumb and index finger, in an immersive haptic-free virtual environment delivered via Oculus head-mounted display. Instantaneous perturbations of object size and distance were randomly applied at 100 ms, 200 ms, or 300 ms (i.e., the moment the start switch—depicted by the solid yellow circle—was released).

Participants reached to grasp virtual objects of different sizes and placed at different distances from the starting position in an immersive hf-VE developed in UNITY (ver. 5.6.1f1, 64-bit, Unity Technologies, San Francisco, CA) and delivered via an Oculus head-mounted display (HMD; Rift DK2, Oculus Inc., Menlo Park, CA; Fig. 1) using HANDoVR (Movement Neuroscience Laboratory, Northeastern University, Boston, MA). An eight-camera motion tracking system (sampling rate: 75 Hz; PPT Studio N^TM^, WorldViz Inc., Santa Barbara, CA) recorded the 3D motion of IRED markers attached to the participants’ wrist (at the center of the segment running between the ulnar and radial styloid processes), and the tips of the thumb and index finger. A pair of IRED markers were attached to the HMD to co-register the participant’s head motion to the virtual environment. Participants viewed the thumb and index fingertips as two 3D spheres (green in color, 0.8 cm diameter) in the hf-VE, reflecting the 3D position of the respective IRED marker. The schedule of trials, virtual renderings of objects, and timing/triggering of perturbations were controlled using custom software developed in C# and Python.

### EMG recordings

EMG activity (in µV) was recorded from the following ten muscles of each participant’s shoulder, arm and hand on the dominant right side. EMG was acquired from the first dorsal interosseous (FDI), flexor digitorum superficialis (FDS), extensor digitorum communis (EDC), extensor indicis (EI), abductor pollicis brevis (APB), extensor pollicis brevis (EPB), biceps brachii (BB), triceps brachii (TB), anterior deltoid (AD), and posterior deltoid (PD).

EMG was recorded using a Delsys TrignoTM wireless EMG system (sampling rate: 1 kHz; Delsys Inc., Natick, MA). Surface EMG sensor bars were attached perpendicular to the muscle fibers over the muscle belly. Excess hair was shaved, and the skin prepped/cleaned with isopropyl alcohol pads before attaching the sensors to reduce skin impedance. Proper positioning of EMG sensors was ensured by physically palpating the muscle during sustained isometric contraction and visual confirmation of the EMG signal. EMG activity during MVC was saved and is included in the dataset. Kinematic and EMG data were synchronized using a 5V digital output (10 ms) sent from Unity and recorded as an analog signal synchronously with EMG.

### Synchronization between EMG data and kinematics data

Details of the synchronization between EMG data and kinematics data are as follows: EMG data were collected using custom software in Matlab to communicate with a Multifunctional I/O Device (NI6255; National Instruments Inc., Austin, TX). Analog data from the Delsys wireless EMG system were streamed to the NI6255 with a known constant 47 ms delay. Kinematic data were collected using C# and Unity-based HANDoVR, described above. HANDoVR software communicated with a National Instrument Multifunctional I/O Device (NI6211). Upon detecting switch release, HANDoVR triggered a 5 V digital (10 ms) output from the NI6211. This digital output was connected to an analog input channel on the NI6255 and recorded into Matlab. EMG and Kinematic data sets were aligned via the start switch trigger recorded digitally in HANDoVR, and the analog reading of the digital output sent from HANDoVR to Matlab. Misalignment of the kinematic data and EMG was constrained to the sampling period of the kinematic data (~11.1 ms), as we were unable to estimate when in the inter-sample period the digital output was sent with respect to when the motion capture sensors were read. Hardware delays were tested to be less than the sampling period of the EMG recording (1 ms).

### Schedule of trials and visual perturbations of object size and distance

Participants were tested in a single experimental session that lasted up to 180 min. Before data collection, participants were allowed to practice the reach-to-grasp task (unperturbed) until they felt comfortable with the task. Then the experiment began, consisting of a total of 960 reach-to-grasp trials, each trial lasting 3.5 s (480 no-perturbation and 480 perturbation trials). The trials were conducted over four sessions of 240 trials each (120 no-perturbation and 120 perturbation trials). The order of no-perturbation and perturbation trials were randomized differently in each session. We ensured that each type of no-perturbation and perturbation trial was evenly distributed across the four sessions. A 5-min break was given after each block and whenever the participant expressed a need to do so.

#### Size perturbation:

Table 1 tabulates the breakdown of 960 trials.

**Table 1:** Visual perturbations of object size.

Condition name	No perturbation	Object size S [cm]	Object distance D [cm]	# Trials	
Small	S	3.5	30	96	
Small-Medium	SM	4.5	30	96	
Medium	M	5.5	30	96	
Medium-Large	ML	6.5	30	96	
Large	L	7.5	30	96	
**Condition name**	**Perturbation**	**Object size S [cm]**	**Object distance D [cm]**	**Perturbation timing [ms]**	**# Trials**
Small→Small-Medium	S→SM	3.5→4.5	30	100, 200, 300	16
Small→Medium	S→M	3.5→4.5	30	100, 200, 300	16
Small→Medium-Large	S→ML	3.5→6.5	30	100, 200, 300	16
Small→Large	S→L	3.5→7.5	30	100, 200, 300	16
Small-Medium**→**Medium	SM→M	4.5→5.5	30	100, 200, 300	16
Small-Medium→Medium-Large	SM→ML	4.5→6.5	30	100, 200, 300	16
Small-Medium→Large	SM→L	4.5→7.5	30	100, 200, 300	16
Medium→Medium-Large	M→ML	5.5→6.5	30	100, 200, 300	16
Medium→Large	M→L	5.5→7.5	30	100, 200, 300	16
Medium-Large→Large	ML→ L	6.5→7.5	30	100, 200, 300	16

The 480 no-perturbation (control) trials were evenly distributed among objects of five different sizes (96 trials per object): small (*w* × *h* × *d* = 3.5 × 8 × 2.5 cm), small-medium (4.5 × 8 × 2.5 cm), medium (5.5 × 8 × 2.5 cm), medium-large (6.5 × 8 × 2.5 cm), and large (7.5 × 8 × 2.5 cm) placed at the same distance of 30 cm from the starting position of the participant’s thumb and index finger. The 96 trials for each object size were evenly distributed across the four blocks (24 trials per block).

The 480 perturbation trials were evenly distributed among ten possible combinations of object size changes such that the object’s width increased from the object’s initial size to a larger size (48 trials per perturbation type). The perturbation types included: small (S) to small-medium (SM), small to medium (M), small to medium-large (ML), small to large (L), small-medium to medium, small-medium to medium-large, small-medium to large, medium to medium-large, medium to large, and medium-large to large (all perturbations from smaller to larger objects). Each perturbation type was applied at three different latencies: 100 ms after movement onset, 200 ms after movement onset, and 300 ms after movement onset, resulting in 16 trials for each perturbation type applied at each of the three timings. The 16 trials for each perturbation type and timing were evenly distributed across the four blocks (four trials per block).

#### Distance perturbation:

Table 2 tabulates the breakdown of 960 trials.

**Table 2:** Visual perturbations of object distance.

Condition name	No perturbation	Object size S [cm]	Object distance D [cm]	# Trials	
Near	N	5.5	20	96	
Near-Middle	NM	5.5	25	96	
Middle	M	5.5	30	96	
Middle-Far	MF	5.5	35	96	
Far	F	5.5	40	96	
**Condition name**	**Perturbation**	**Object size S [cm]**	**Object distance D [cm]**	**Perturbation timing [ms]**	**# Trials**
Near→Near-Middle	N→NM	5.5	20→25	100, 200, 300	16
Near→Middle	N→M	5.5	20→30	100, 200, 300	16
Near→Middle-Far	N→MF	5.5	20→35	100, 200, 300	16
Near→Far	N→F	5.5	20→40	100, 200, 300	16
Near-Middle**→**Middle	NM→M	5.5	25→30	100, 200, 300	16
Near-Middle→Middle-Far	NM→MF	5.5	25→35	100, 200, 300	16
Near-Middle→Far	NM→F	5.5	25→40	100, 200, 300	16
Middle→Middle-Far	M→MF	5.5	30→35	100, 200, 300	16
Middle→Far	M→F	5.5	30→40	100, 200, 300	16
Middle-Far→Far	MF→F	5.5	35→40	100, 200, 300	16

The 480 no-perturbation (control) trials were evenly distributed among objects (all 5.5 × 8 × 2.5 cm) placed at five different distances (96 trials per object): near (20 cm), near-middle (25 cm), middle (30 cm), middle-far (35 cm), and far (40 cm). The 96 trials for each object distance were evenly distributed across the four blocks (i.e., 24 trials per block).

The 480 perturbation trials were evenly distributed among ten possible combinations of object distance changes such that the object’s distance increased from the object’s initial location to a farther location (48 trials per perturbation type). The perturbation types included: near (N) to near-middle (NM), near to middle (M), near to middle-far (MF), near to far (F), near-middle to middle, near-middle to middle-far, near-middle to far, middle to middle-far, middle to far, and middle-far to far (all permutations from closer to farther distances). Each perturbation type was applied at three different latencies: 100 ms after movement onset, 200 ms after movement onset, and 300 ms after movement onset, resulting in 16 trials for each perturbation type applied at each of the three timings. The 16 trials for each perturbation type and timing were evenly distributed across the four blocks (four trials per block). The reach-to-grasp animation of representative conditions (control, size and distance perturbations with 100 and 300 ms latencies) is available on the Figshare^43^.

### Procedures and instructions to participants

Each participant was seated in a chair with their right hand placed on a table in front of them (Fig. 1). At the start position, the thumb and index finger straddled a 1.5 cm wide wooden peg located 12 cm in front and 24 cm to the right of the sternum, with the thumb depressing a start switch. Lifting the thumb off the switch marked movement onset. A digital transistor–transistor logic (TTL) connected to the start switch was used to synchronize kinematic and EMG recordings. In each trial, the following events occurred: (1) Participants depressed the start switch to begin the trial. (2) The object appeared in hf-VE, oriented at a 75° angle along the vertical axis to minimize excessive wrist extension during reach-to-grasp. (3) After 1 s, an auditory cue—a beep—signaled the participants to reach for, grasp, and lift the object with 1.2 cm combined error margin^44^. The object was considered to have been grasped when both 3D spheres (reflecting the tips of the thumb and index finger) had come in contact with the lateral surfaces of the virtual object. (4) Once grasp of the virtual object was detected, the object changed color from blue to red and a ‘click’ sound was presented. (5) Participants lifted and raised each object briefly before returning their hand to the starting position, after which the next trial began.

Instructions to the participant were: “Each trial will start once the thumb depresses the start switch (the correct initial position of the hand was demonstrated). Following the beep, reach-to and grasp the narrow sides of the object between the thumb and index finger using a pincer grip (demonstrated). When the object is grasped, it will turn from blue to red and a ‘click’ sound will be presented. Lift the object briefly until the object disappears, and return your hand to the start position. On some trials, the object may change “size” or “position”, requiring you to adjust your movements. A break would be provided after each block of 240 trials but you may rest at any point between trials within a block by not depressing the start switch to begin the next trial. Do you have any questions?”.

### Data processing and kinematic feature extraction

The raw data included the *x*, *y*, and *z* marker positions of the wrist, thumb, and index finger positions with associated timestamps (75 Hz; this raw data is not provided in the data records). All position data were analyzed offline using custom Matlab codes. The time-series data for each trial were cropped from movement onset (the moment the switch was released) to movement offset (the moment the collision detection criteria were met) and resampled at 100 Hz using the interp1() function in Matlab. Transport distance (the straight-line distance of the wrist marker from the starting position in the transverse plane) and aperture (the straight-line distance between the thumb and index finger markers in the transverse plane) were computed for each trial. The first and second derivatives of transport displacement and aperture were computed to obtain the velocity and acceleration profiles for kinematic feature extraction. A 6 Hz, fourth-order low-pass Butterworth filter was applied on all time-series. Trails in which participants did not move, were delayed in moving, or had inappropriate movements were excluded from the database (i.e., bad trials). Link to the GitHub repository of custom code used to generate the data is available under Code Availability section.

**Fig. 2:**
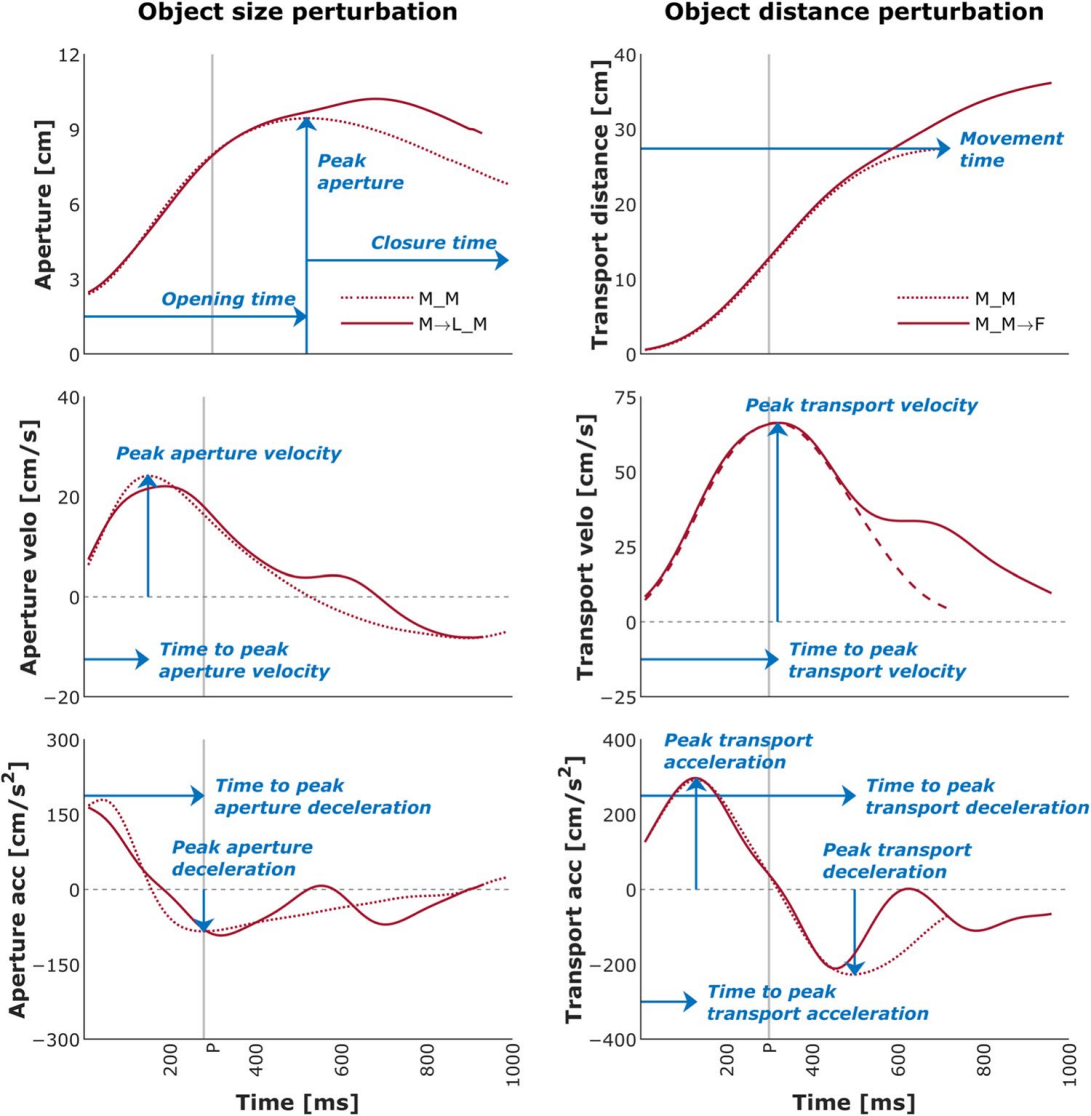
Mean temporal profiles of transport and aperture kinematics for the control (no perturbation) and a size/ditsance-perturbation condition (perturbation applied at 300 ms after movement onset) for a representative participant. Blue arrows indicate the kinematic features listed in Table 3. Light gray vertical line indicates the timing of perturbation—P. Conditions: M_M: Medium:Middle; M_M→F: Medium:Middle→Far; M_LM: Medium→Large:Middle.

For each trial, the following kinematic features, units in parentheses (Table 3), were extracted using the filtered time series data (Fig. 2):

- Movement time [ms]—duration from movement onset to movement offset.
- Peak transport velocity [cm/s]—maximum velocity of the wrist marker.
- Time to peak transport velocity [ms]—time from movement onset to maximum velocity of the wrist marker.
- Peak transport acceleration [cm/s^2^]—maximum acceleration of the wrist marker.
- Time to peak transport acceleration [ms]—time from movement onset to maximum acceleration of the wrist marker.
- Peak transport deceleration [cm/s^2^]—maximum deceleration of the wrist marker.
- Time to peak transport deceleration [ms]—time from movement onset to maximum deceleration of the wrist marker.
- Peak aperture [cm]—maximum distance between the fingertip markers. Peak aperture also marked the initiation of closure or Closure Onset (henceforth, CO) and which we refer to as the aperture at CO.
- Peak aperture velocity [cm/s]—maximum velocity of the aperture.
- Time to peak aperture velocity [ms]—time from movement onset to maximum velocity of the aperture before CO.
- Peak aperture deceleration [cm/s^2^]—maximum deceleration of the aperture before CO.
- Time to peak aperture deceleration [ms]—time from movement onset to maximum deceleration of the aperture before CO.
- Opening time [ms]—duration from movement onset to peak aperture.
- Closure time [ms]—duration from CO to movement offset.
- Opening distance [cm]—distance between the wrist’s position at movement onset and the wrist’s position at CO.
- Closure distance [cm]—distance between the wrist’s position at CO and the object’s center.
- Transport velocity at CO [cm/s]—velocity of the wrist marker at the time of CO.
- Transport acceleration at CO [cm/s^2^]—acceleration of the wrist marker at the time of CO.
- Peak closure velocity [cm/s]—minimum velocity of the aperture after CO.
- Peak closure deceleration [cm/s^2^]—maximum deceleration of the aperture after CO.

**Table 3:** Order of kinematic features (from top to bottom).

Feature name in data record	Kinematic feature	Unit
MT	Movement time	ms
Peak_TV	Peak transport velocity	cm/s
T_Peak_TV	Time to peak transport velocity	ms
Peak_TA	Peak transport acceleration	cm/s^2^
T_Peak_TA	Time to peak transport acceleration	ms
Peak_TD	Peak transport deceleration	cm/s^2^
T_Peak_TD	Time to peak transport deceleration	ms
Peak_A	Peak aperture	cm
Peak_AV	Peak aperture velocity	cm/s
T_Peak_AV	Time to peak aperture velocity	ms
Peak_AD	Peak aperture deceleration	cm/s^2^
T_Peak_AD	Time to peak aperture deceleration	ms
OT	Opening time	ms
CT	Closure time	ms
OD	Opening distance	cm
CD	Closure distance	cm
TV_CO	Transport velocity at CO	cm/s
TA_CO	Transport acceleration at CO	cm/s^2^

Before data collection, the maximal voluntary contraction (MVC) of each muscle was obtained. Muscle activation was recorded during each reach-to-grasp movement for 3.5 s. The data files accompanying this dataset contain MVCs and raw EMG beginning 500 ms before movement onset. EMG data from one participant (P6 - size perturbation only) were not saved correctly due to technical issues.

## Data Records

**Fig. 3:**
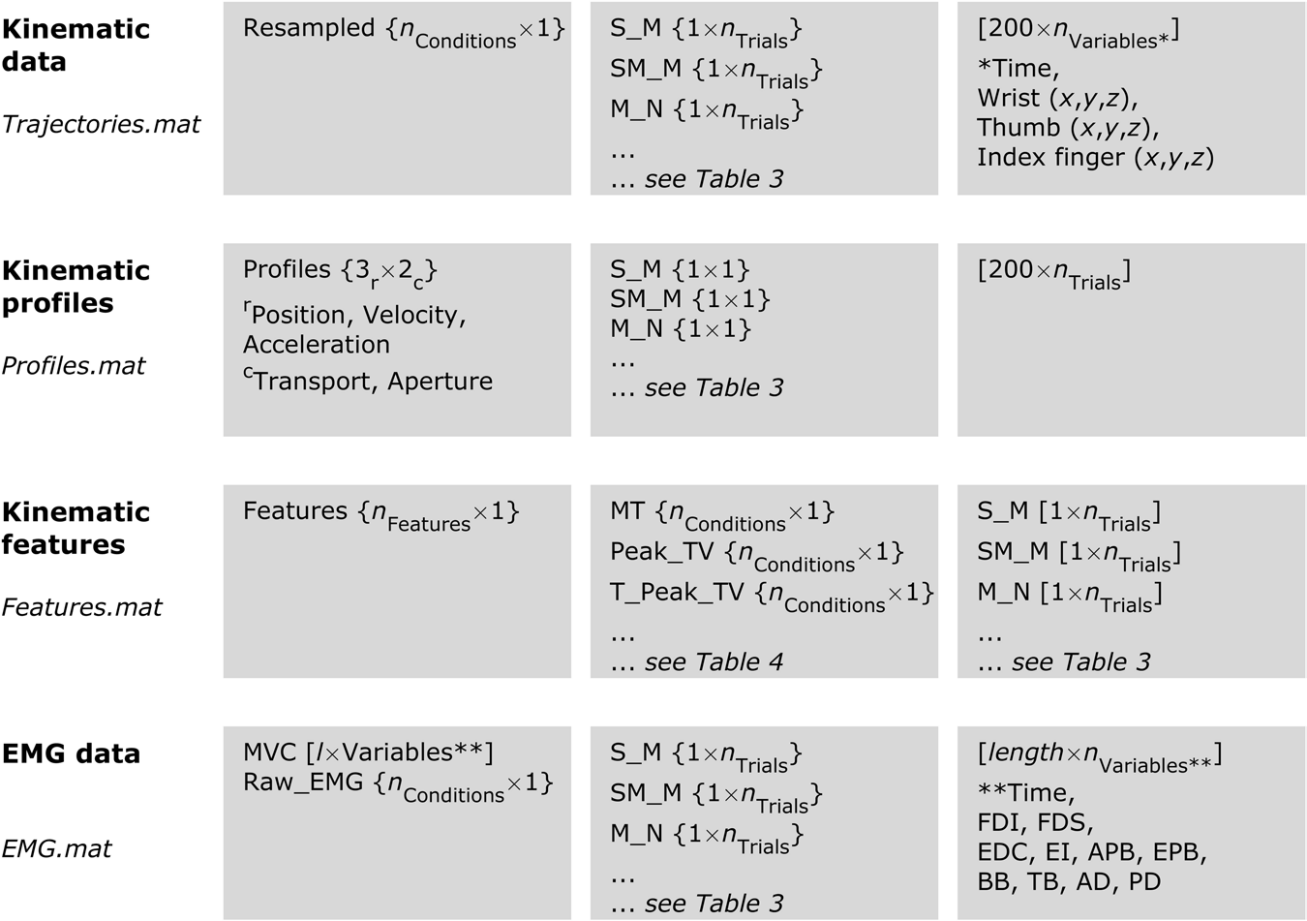
Matlab structure in which the data are saved.

All data is made available using Figshare^45^. All 10 participants for each type of perturbation have been identified using alphanumeric format P# in the folders “SizePert” and “DistPert” for object size and distance perturbation, respectively. The deidentified participant information (sex, age, body mass, and height) is stored in the Excel file named “Participants”. Kinematic and EMG data have been grouped into subject-specific folders, each folder bearing the participant’s alphanumeric code (e.g., P5 for the fifth participant). Within each participant folder, there are five.mat files: 1) Raw_Data; 2) Resampled data; 2) Kinematic profiles of position, velocity, and acceleration for both the transport and aperture; 3) Kinematic features; 5) MVC and raw EMG. Figure 3 illustrates the Matlab structure in which files 2–5 are saved, with the row and column vectors shown in red (also see Tables 3 and 4).

- *Raw_Data.mat*—this file contains nine arrays:

- Trial_Number—1 × 960 cell array with each cell containing the trial number of the respective trial in the same order in which that trial was conducted.
- Trial_Status—1 × 960 cell array with each cell containing information about whether that trial was a bad trial, that is, the one in which the participant did not move, was delayed in moving or had inappropriate movement.
- Condition_Names—1 × 960 string array of condition names indicating the size and distance of object, perturbation, and the timing of perturbation (see Table 4).
- Variable_Names—1 × 10 string array of variable names and measurement units corresponding to each column in the data matrix for each trial in the ‘Raw_Trajectories’ array.
- Raw_Trajectories—1 × 960 cell array with *n*^th^ column corresponding to *n*^th^ trial. Each cell of this array contains a *t* × 10 matrix, where *t* is the number of samples captured by the motion capture system at 75 Hz for the respective trial. The next nine columns of this matrix correspond to the time stamp and the *x*-, *y*-, and *z*- coordinates of the wrist, thumb, and index finger markers (see Variable_Names).
- Onset—1 × 960 numeric array with each cell containing the timing of movement onset for the respective trial based on switch release.
- Offset—1 × 960 numeric array with each cell containing the timing of movement offset for the respective trial based on collision detection.
- Corrected_Onset—1 × 960 numeric array with each cell containing the manually-selected timing of movement onset for the respective trial used to crop data during post-processing. Each trial was visually inspected in the post-processing stage. Movement onset was corrected if the onset was delayed (i.e., exceeded 3% of peak aperture) or untimely marked (i.e., there was no change in aperture for >2 samples). Onset was corrected only for control trials.
- Corrected_Offset—1 × 960 numeric array with each cell containing the manually-selected timing of movement offset for the respective trial used to crop data during post-processing. Each trial was visually inspected in the post-processing stage. Movement offset was corrected if the offset did not fall and remain below 3% of transport velocity of peak transport velocity.

- *Trajectories.mat*—this file contains four arrays:

- Resampled—35 × 1 cell array with each row corresponding to a different condition (see Condition_Names). Each cell of this array contains a 1 × *n*_Trials_ cell array (*n*_Trials_ = number of trials) with each cell containing data for an individual trial in 200 × 10 matrix. The columns of this matrix correspond to the time stamp and the *x*-, *y*-, and *z*- coordinates of the wrist, thumb, and index finger markers (see Variable_Names).
- Condition_Names—35 × 1 string array of condition names indicating the size and distance of object, perturbation, and the timing of perturbation (see Table 4).
- Variable_Names—1 × 10 string array of variable names and measurement units corresponding to each column in the data matrix for each trial.
- Final_Object_Location—35 × 1 cell array with each cell containing the x-, y-, and z- coordinates of the final object position for each of the 35 conditions.

- Profiles.mat—this file contains three arrays:

- Profiles—3 × 2 cell array with the three rows corresponding to the position, velocity, and acceleration and the two columns corresponding to the transport and aperture. Each cell of this array contains a 35 × 1 cell array with each row corresponding to a different condition (see Condition_Names). Each cell of this array contains a 200 × *n*_Trials_ matrix (*n*_Trials_ = number of trials) with each column containing data for an individual trial.
- Variable_Names—3 × 2 string array of variable names.
- Condition_Names—35 × 1 string array of condition names indicating the size and distance of object, perturbation, and the timing of perturbation (see Table 4).

- Features.mat—this file contains three arrays:

- Features—18 × 1 cell array with each row corresponding to a different kinematic feature (see Feature_Names). Each cell of this array contains a 35 × 1 cell array with each row corresponding to a different condition (see Condition_Names). Each cell of this array is a 1 × *n*_Trials_ matrix with the columns containing data for individual trials.
- Feature_Names—18 × 1 string array with the names and measurement units of kinematic features (see Table 3).
- Condition_Names—35 × 1 string array of condition names indicating the size and distance of object, perturbation, and the timing of perturbation (see Table 4).

- EMG.mat—this file contains five arrays:

- MVC—4000 × 11 matrix with the columns corresponding to the time stamp and EMG activity in each of the ten recorded muscles (see Variable_Names).
- Variable_Names—1 × 11 string array of variable names and measurement units corresponding to each column in the data matrix for each trial.
- Raw_EMG—35 × 1 cell array with each row corresponding to a different condition (see Condition_Names). Each cell of this array contains a 1 × *n*_Trials_ cell array (*n*_Trials_ = number of trials) with each cell containing data for an individual trial in a 4000 × 11 matrix. The columns of this matrix correspond to the time stamp and EMG activity in the ten recorded muscles (see Variable_Names).
- Condition_Names—35 × 1 string array of condition names indicating the size and distance of object, perturbation, and the timing of perturbation (see Table 4).
- Movement_Time—18 × 1 cell array with each row corresponding to a different kinematic feature (see Feature_Names). Each cell of this array contains a 35 × 1 cell array with each row corresponding to a different condition (see Condition_Names). Each cell of this array is a 1 × *n*_Trials_ matrix with the columns containing movement time [ms] for individual trials.

**Table 4:** Order of conditions (from left to right in data files).

Size perturbation		
	Condition name in data record*	Condition^†^
No perturbation	S_M	Small:Middle
	SM_M	Small-Medium:Middle
	M_M	Medium:Middle
	ML_M	Medium-Large:Middle
	L_M	Large:Middle
Perturbation	StoSM_M_100/200/300	Small→Small-Medium (100, 200, & 300 ms)
	StoM_M_100/200/300	Small→Medium (100, 200, & 300 ms)
	StoML_M_100/200/300	Small→Medium-Large (100, 200, & 300 ms)
	StoL_M_100/200/300	Small→Large (100, 200, & 300 ms)
	SMtoM_M_100/200/300	Small-Medium→Medium (100, 200, & 300 ms)
	SMtoML_M_100/200/300	Small-Medium→Medium-Large (100, 200, & 300 ms)
	SMtoL_M_100/200/300	Small-Medium→Large (100, 200, & 300 ms)
	MtoML_M_100/200/300	Medium→Medium-Large (100, 200, & 300 ms)
	MtoL_M_100/200/300	Medium→Large (100, 200, & 300 ms)
	MLtoL_M_100/200/300	Medium-Large→Large (100, 200, & 300 ms)
**Distance perturbation**		
	Condition name in data record*	**Condition**^**†**^
No perturbation	M_N	Medium:Near
	M_NM	Medium:Near-Middle
	M_M	Medium:Middle
	M_MF	Medium:Middle-Far
	M_F	Medium:Far
Perturbation	M_NtoNM_100/200/300	Near→Near-Middle (100, 200, & 300 ms)
	M_NtoM_100/200/300	Near→Middle (100, 200, & 300 ms)
	M_NtoMF_100/200/300	Near→Middle-Far (100, 200, & 300 ms)
	M_NtoF_100/200/300	Near→Far (100, 200, & 300 ms)
	N_NMtoM_100/200/300	Near-Middle→Middle (100, 200, & 300 ms)
	M_NMtoMF_100/200/300	Near-Middle→Middle-Far (100, 200, & 300 ms)
	M_NMtoF_100/200/300	Near-Middle→Far (100, 200, & 300 ms)
	M_MtoMF_100/200/300	Middle→Middle-Far (100, 200, & 300 ms)
	M_MtoF_100/200/300	Middle→Far (100, 200, & 300 ms)
	M_MFtoF_100/200/300	Middle-Far→Far (100, 200, & 300 ms)

*Format: Object size_Object distance_Perturbation timing; arrows indicate perturbation.

^†^Parenthesized values indicate perturbation timin.

## Technical Validation

### Kinematic data: Effect of perturbations on reach-grasp coordination

**Fig. 4:**
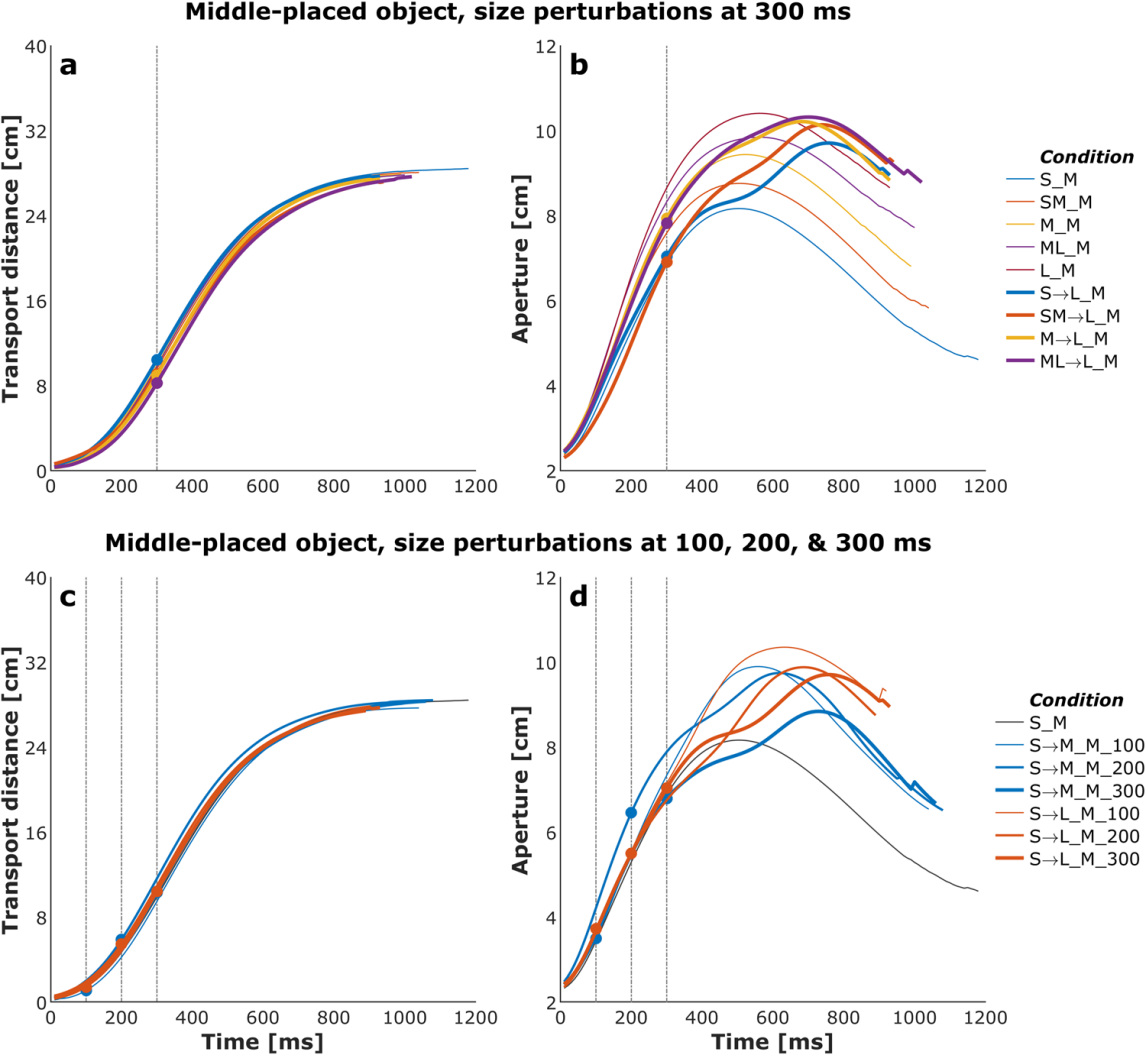
Plots of mean transport distance and aperture for the control (no perturbation) and a selected set of size-perturbation conditions for a representative participant. (**a**) Transport distance, perturbation applied at 300 ms after movement onset. (**b**) Aperture, perturbation applied at 300 ms after movement onset. (**c**) Transport distance, perturbation applied at 100 ms, 200 ms, or 300 ms after movement onset. (**d**) Aperture, perturbation applied at 100 ms after movement onset, 200 ms after movement onset, or 300 ms after movement onset. Legend format: Object Size_Object Distance; arrows indicate perturbation of object size. Gray vertical dashed-dotted lines and solid circles indicate the timing of the perturbations.

**Fig. 5:**
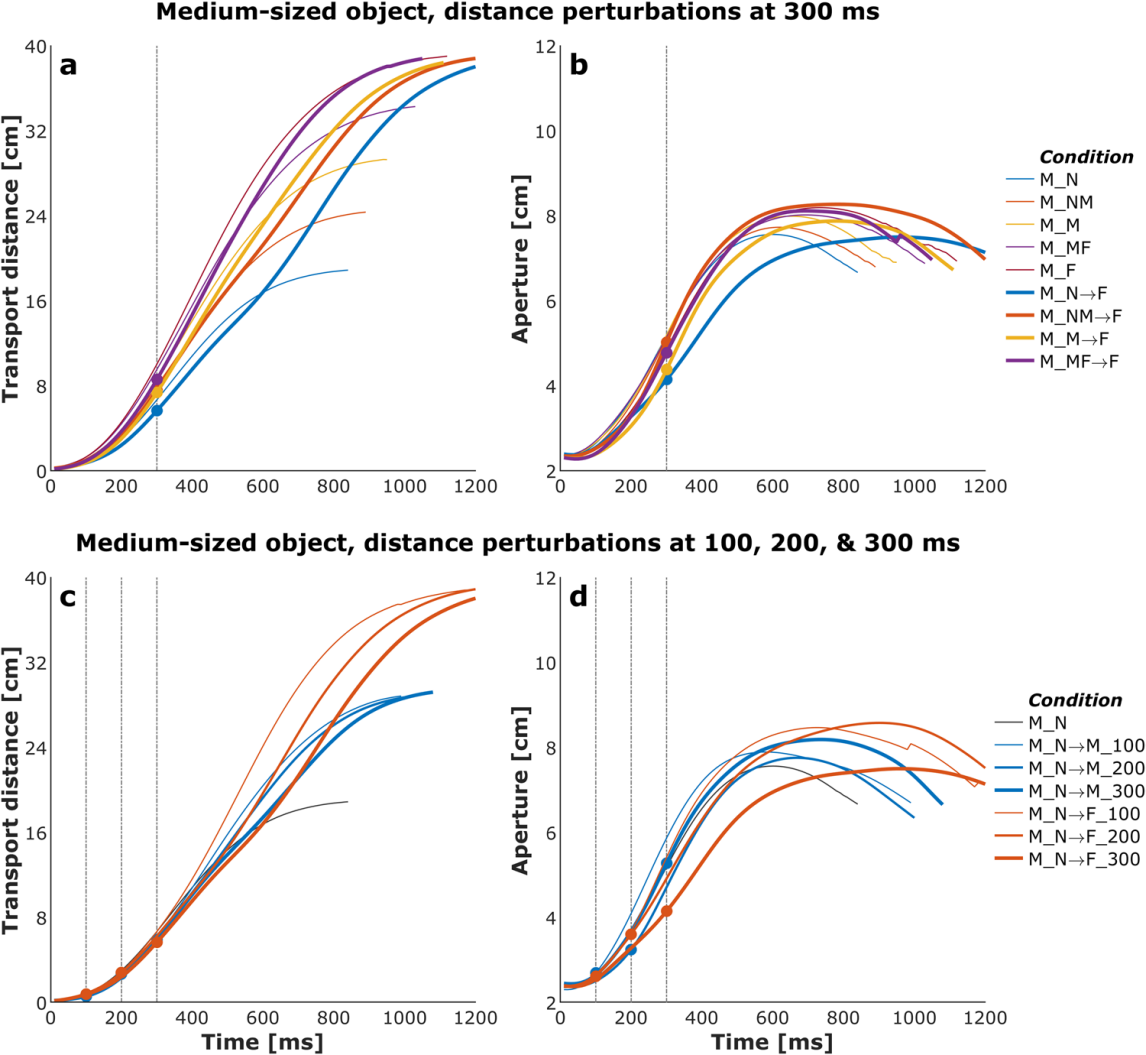
Plots of mean transport distance and aperture for the control (no perturbation) and a selected set of distance-perturbation conditions for a representative participant. (**a**) Transport distance, perturbation applied at 300 ms after movement onset. (**b**) Aperture, perturbation applied at 300 ms after movement onset. (**c**) Transport distance, perturbation applied at 100 ms, 200 ms, or 300 ms after movement onset. (**d**) Aperture, perturbation applied at 100 ms after movement onset, 200 ms after movement onset, or 300 ms after movement onset. Legend format: Object Size_Object Distance; arrows indicate perturbation of object size. Solid circles indicate the timing of the perturbations.

**Fig. 6:**
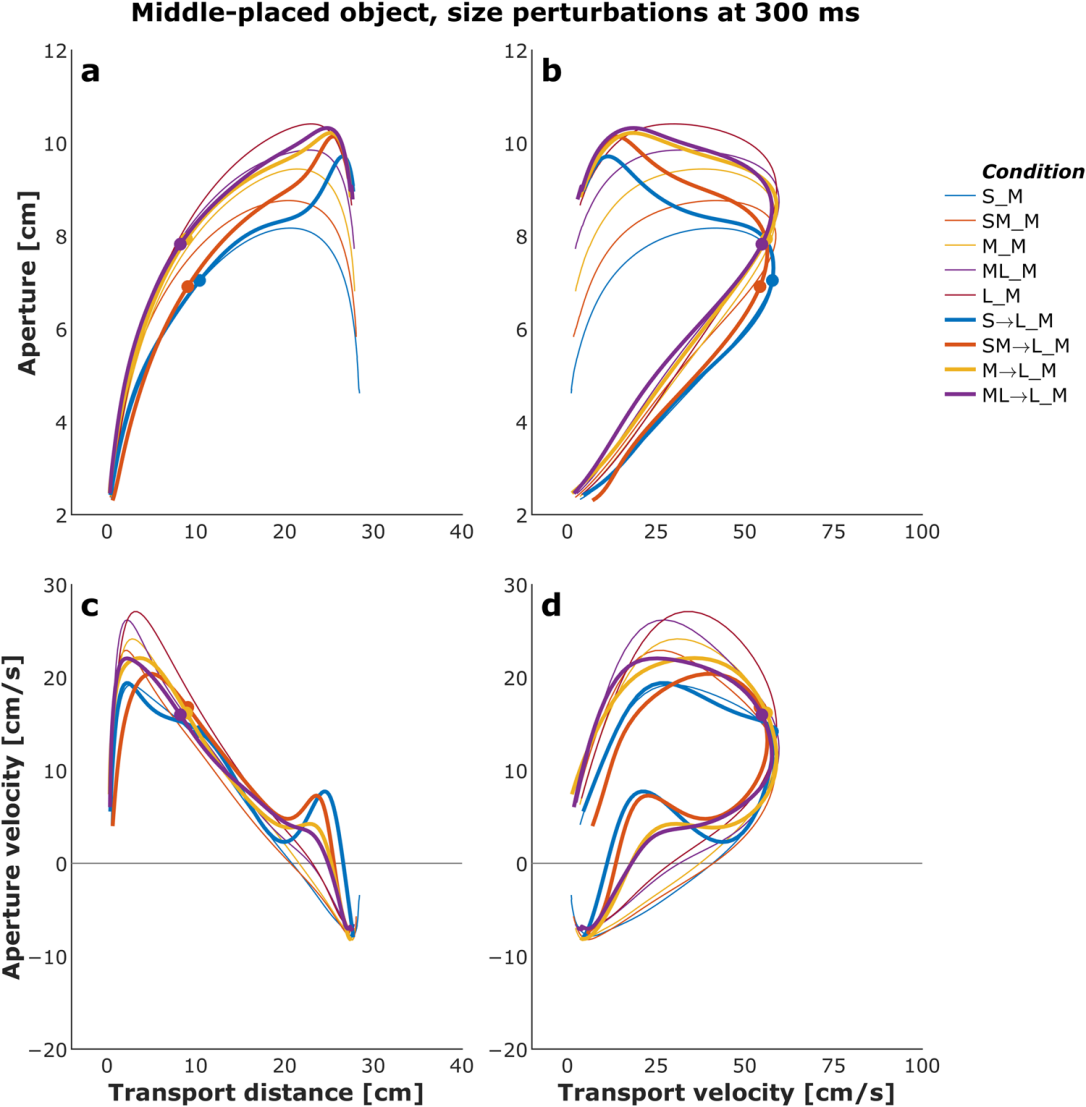
Phase plots of mean transport and aperture kinematics for the control (no perturbation) and a selected set of size-perturbation conditions (perturbation applied at 300 ms after movement onset) for a representative participant. (**a**) Aperture versus transport distance. (**b**) Aperture versus transport distance. (**c**) Aperture velocity versus transport velocity. (**d**) Aperture velocity versus transport velocity. Each profile describes only the first 1000 ms of movement. Legend format: Object Size_Object Distance; arrows indicate perturbation of object size. Solid circles indicate the timing of the perturbations.

**Fig. 7:**
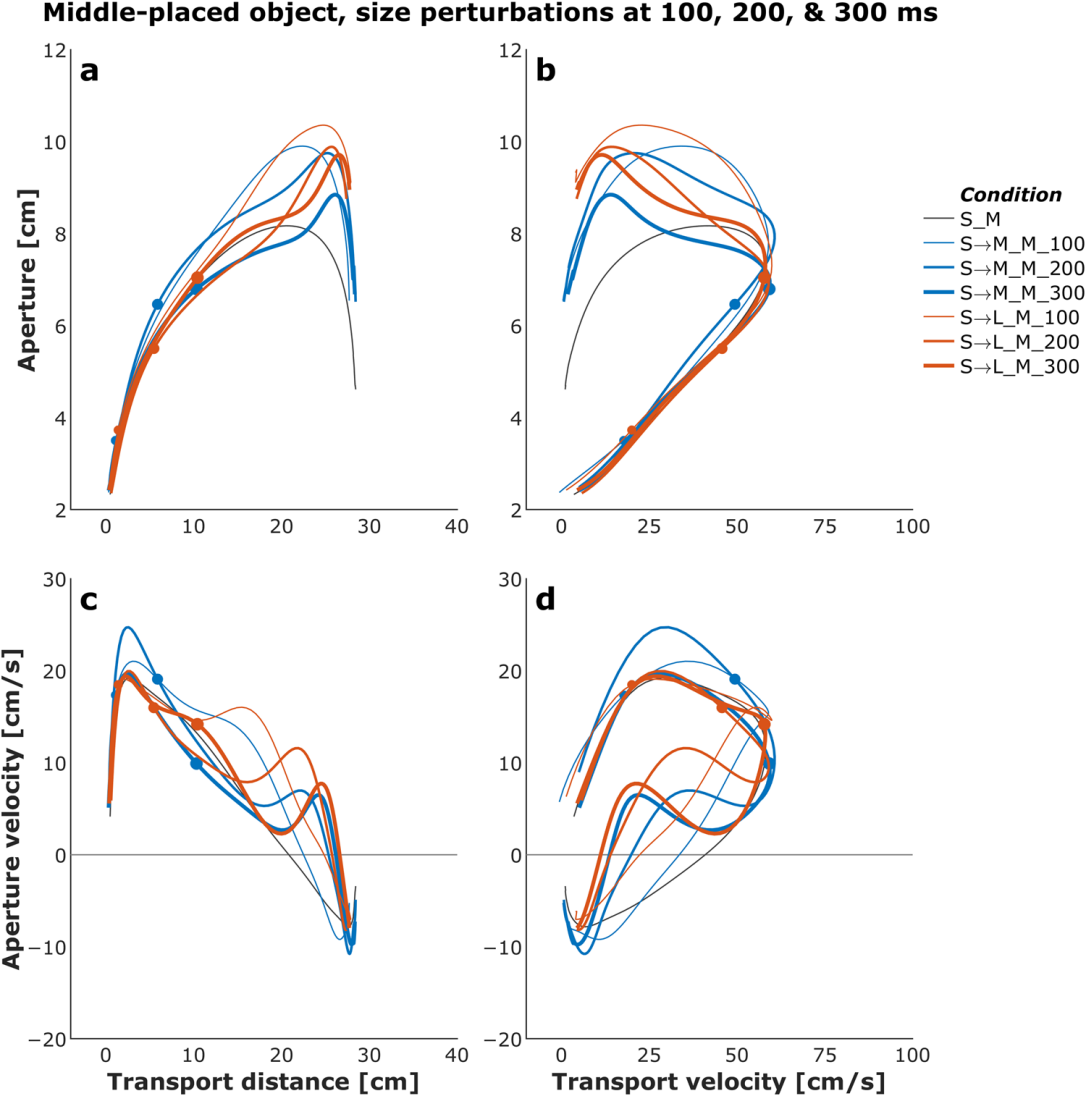
Phase plots of mean transport and aperture kinematics for the control (no perturbation) and a selected set of size-perturbation conditions (perturbation applied at 100 ms, 200 ms, or 300 ms after movement onset) for a representative participant. (**a**) Aperture versus transport distance. (**b**) Aperture versus transport distance. (**c**) Aperture velocity versus transport velocity. (**d**) Aperture velocity versus transport velocity. Each profile describes only the first 1000 ms of movement. Legend format: Object Size_Object Distance; arrows indicate perturbation of object size. Gray vertical dashed-dotted lines and solid circles indicate the timing of the perturbations.

**Fig. 8:**
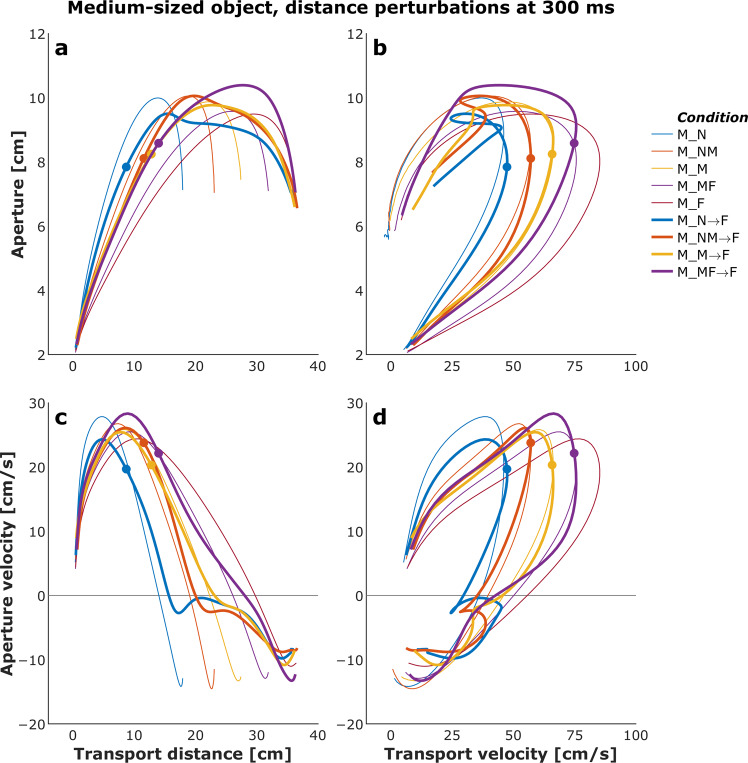
Phase plots of mean transport and aperture kinematics for the control (no perturbation) and a selected set of distance-perturbation conditions (perturbation applied at 300 ms after movement onset) for a representative participant. (**a**) Aperture versus transport distance. (**b**) Aperture versus transport velocity. (**c**) Aperture velocity versus transport velocity. (**d**) Aperture velocity versus transport velocity. Each profile describes only the first 1000 ms of movement. Legend format: Object Size_Object Distance; arrows indicate perturbation of object distance. Solid circles indicate the timing of the perturbations.

**Fig. 9:**
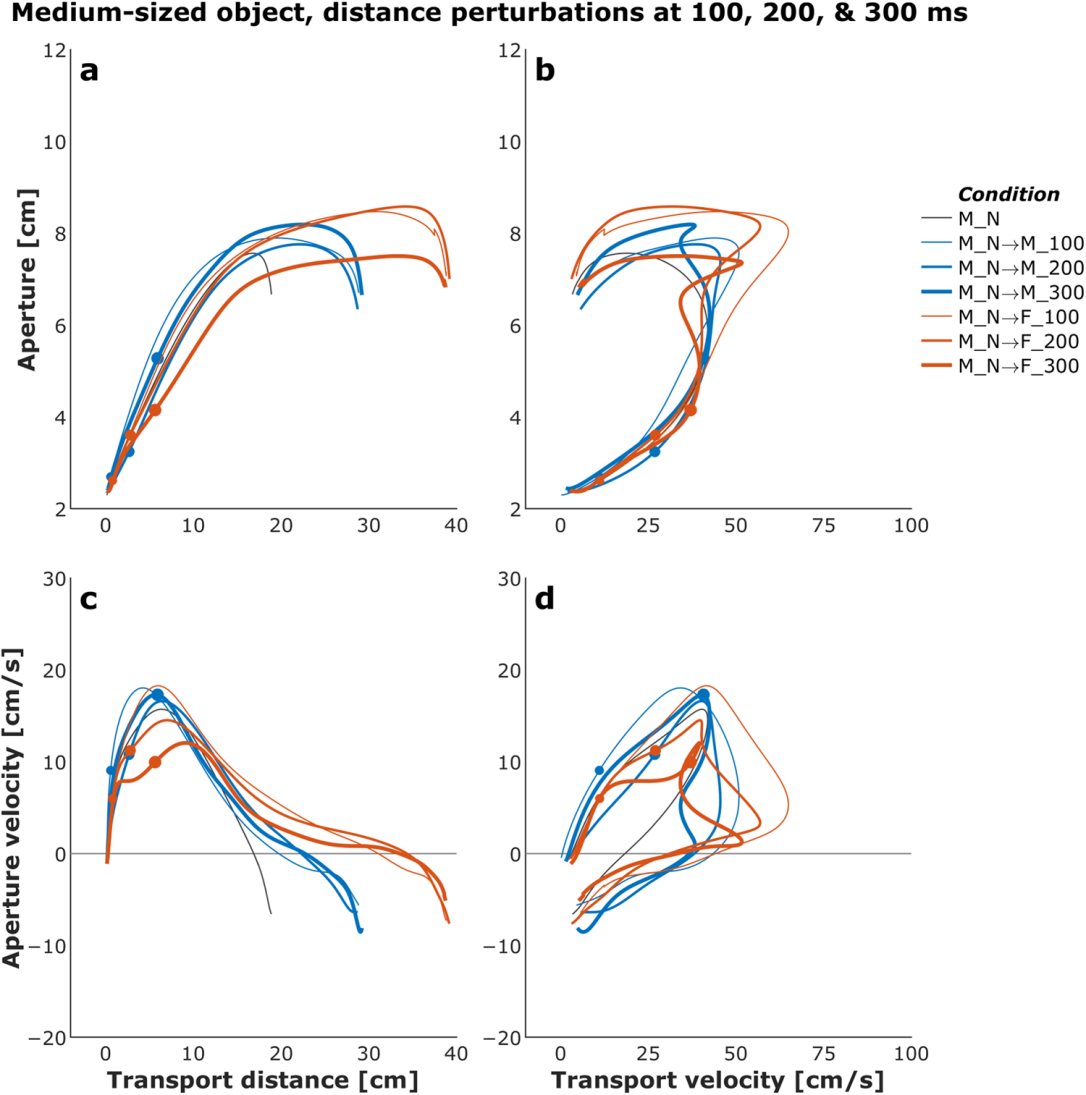
Phase plots of mean transport and aperture kinematics for the control (no perturbation) and a selected set of distance-perturbation conditions (perturbation applied at 100 ms, 200 ms, or 300 ms after movement onset) for a representative participant. (**a**) Aperture versus transport distance. (**b**) Aperture versus transport velocity. (**c**) Aperture velocity versus transport velocity. (**d**) Aperture velocity versus transport velocity. Each profile describes only the first 1000 ms of movement. Legend format: Object Size_Object Distance; arrows indicate perturbation of object distance. Solid circles indicate the timing of the perturbations.

**Fig. 10:**
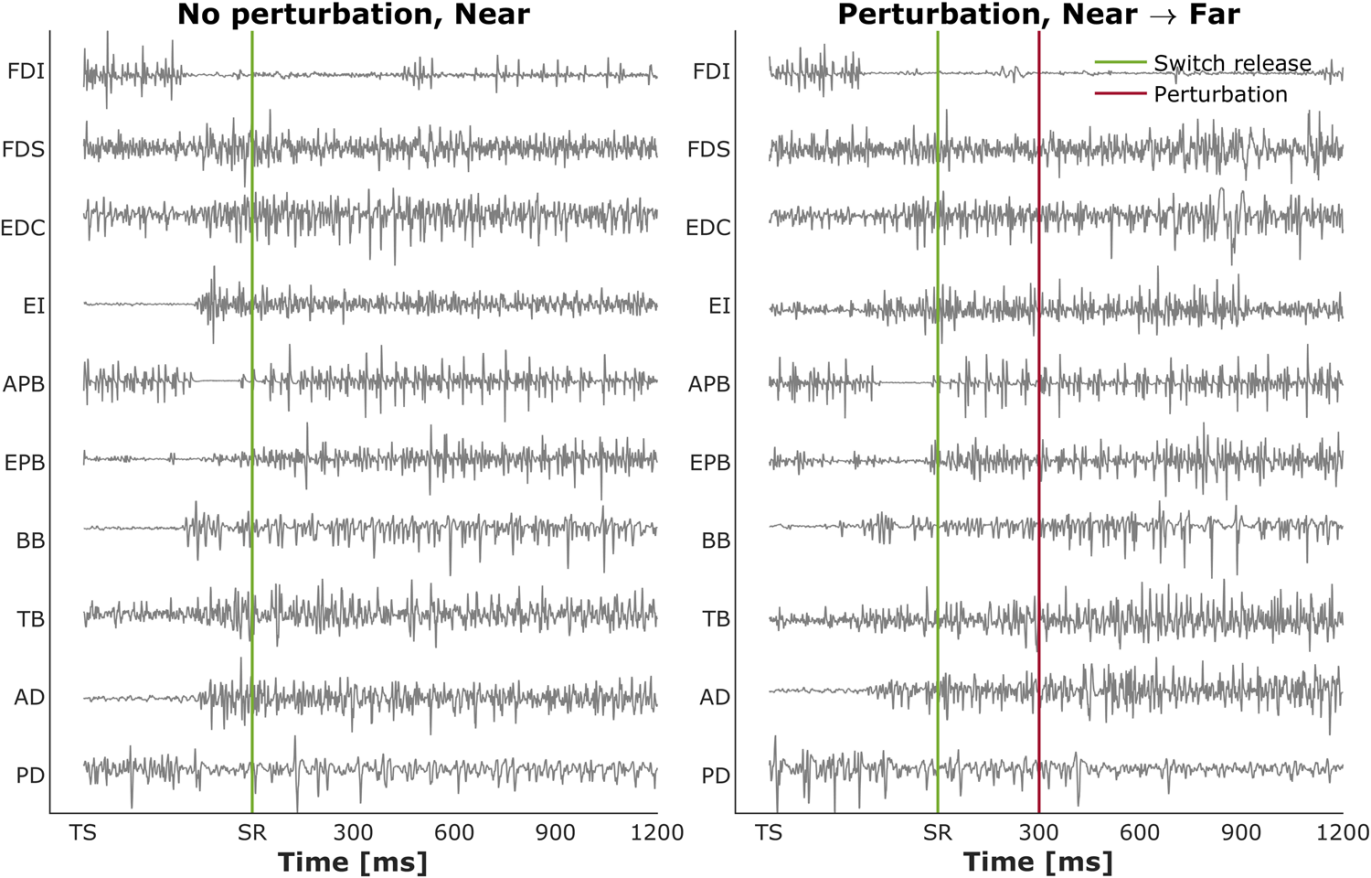
Normalized EMG for each of the 10 muscles in the control (no perturbation) and a distance-perturbation condition (perturbation applied at 300 ms after movement onset) for a representative trial. ‘TS:’ trial start; ‘SR:’ switch release.

Figures 4 and 5 show plots of the mean transport distance and aperture for the control (no perturbation) and a selected set of size- and distance-perturbation conditions, respectively, for a representative participant. Figures 6–9 describe phase plots of mean transport and aperture kinematics for the control and a selected set of size- and distance- perturbation conditions (perturbations applied at 100 ms, 200 ms, and 300 ms after movement onset) for a representative participant. As we presented in our previous work^46^, phase plots allow to distinguish the three phases of the reach-to-grasp coordination (i) *Initiation Phase*, which includes the initial acceleration of transport velocity and the first half of the hand opening, which begins with the rapid opening of the thumb and index finger. (ii) *Shaping Phase*, which begins at maximum transport velocity. It includes the first half of transport deceleration and the second half of the hand opening, which ends when the maximum aperture is achieved, marking the initiation of closure or closure onset, CO. (iii) *Closure Phase*, which includes the second half of transport deceleration and lasts until the object is grasped. Finally, Fig. 10 shows normalized EMG for each of the 10 muscles in the control and a distance-perturbation condition (perturbation applied at 300 ms after movement onset) for a representative trial. These figures provide a glimpse into the qualitative effects of visual perturbations of object size and distance on reach-grasp coordination.

It has been firmly established that the aperture component of reach-to-grasp movement is influenced by the object’s physical dimensions, while the transport component remains relatively unaffected by changes in object size^8,47^. In contrast, the transport component of reach-to-grasp movement is influenced by the object’s spatial location (i.e., the distance from the observer) and precision requirements due to object size, while the aperture component remains relatively unaffected by changes in object distance^7,48^. Hence, visual perturbations of object size evoke online adjustments in grasp aperture, and visual perturbations of object distance evoke online adjustments in transport velocity. Accordingly, to ensure all applied visual perturbations of object size and distance influenced the aperture and transport components in known ways, it was examined whether the size and distance perturbations influenced the peak aperture and peak transport velocity. To this end, movement time, peak aperture, and peak transport velocity were compared between each of the thirty size and distance perturbation conditions and the respective unperturbed condition (e.g., peak aperture for S→SM, S→M, S→ML, S→L each was compared to peak aperture for the S condition). Consistent with the findings from past studies conducted in the real world^49^ and VE^44,46,50^, movement time linearly scaled to object size and distance in the control conditions. The smaller the object the longer the reach-to-grasp movement (L: 977 ms, ML: 1019 ms, M: 1037 ms, SM: 1053 ms S: 1134 ms; rm-ANOVA: F_4,36_ = 24, p < 0.001; Fig. 4), and the farther the object the longer the reach-to-grasp movement (N: 897 ms, NM: 942 ms, M: 984 ms, MF: 1039 ms, F: 1095 ms; F_4,36_ = 49.7, p < 0.001; Fig. 5). As typically observed of reach-to-grasp movements^8,46,47^, peak aperture linearly scaled to object size (S: 8.6 cm, SM: 9.2 cm, M: 9.7 cm, ML: 10.2 cm, L: 10.8 cm; F_4,36_ = 159, p < 0.001; Fig. 6) and showed expected increase with perturbation of object size (Figs. 6 and 7). Similarly, respecting known trends^7,48,49^, peak transport velocity linearly scaled to object distance (N: 49.7 cm/s, NM: 59.3 cm/s, M: 68.7 cm/s, MF: 77.2 cm/s, F: 85.3 cm/s; F_4,36_ = 304.4, p < 0.001; Fig. 8) and showed expected increase with perturbation of object distance (Figs. 8 and 9). Importantly, final aperture was always scaled to object size. These trends provide a strong validation of the expected responses to perturbations of object size and distance during reach-to-grasp.

### EMG data: Spectral properties of EMG signals

**Fig. 11:**
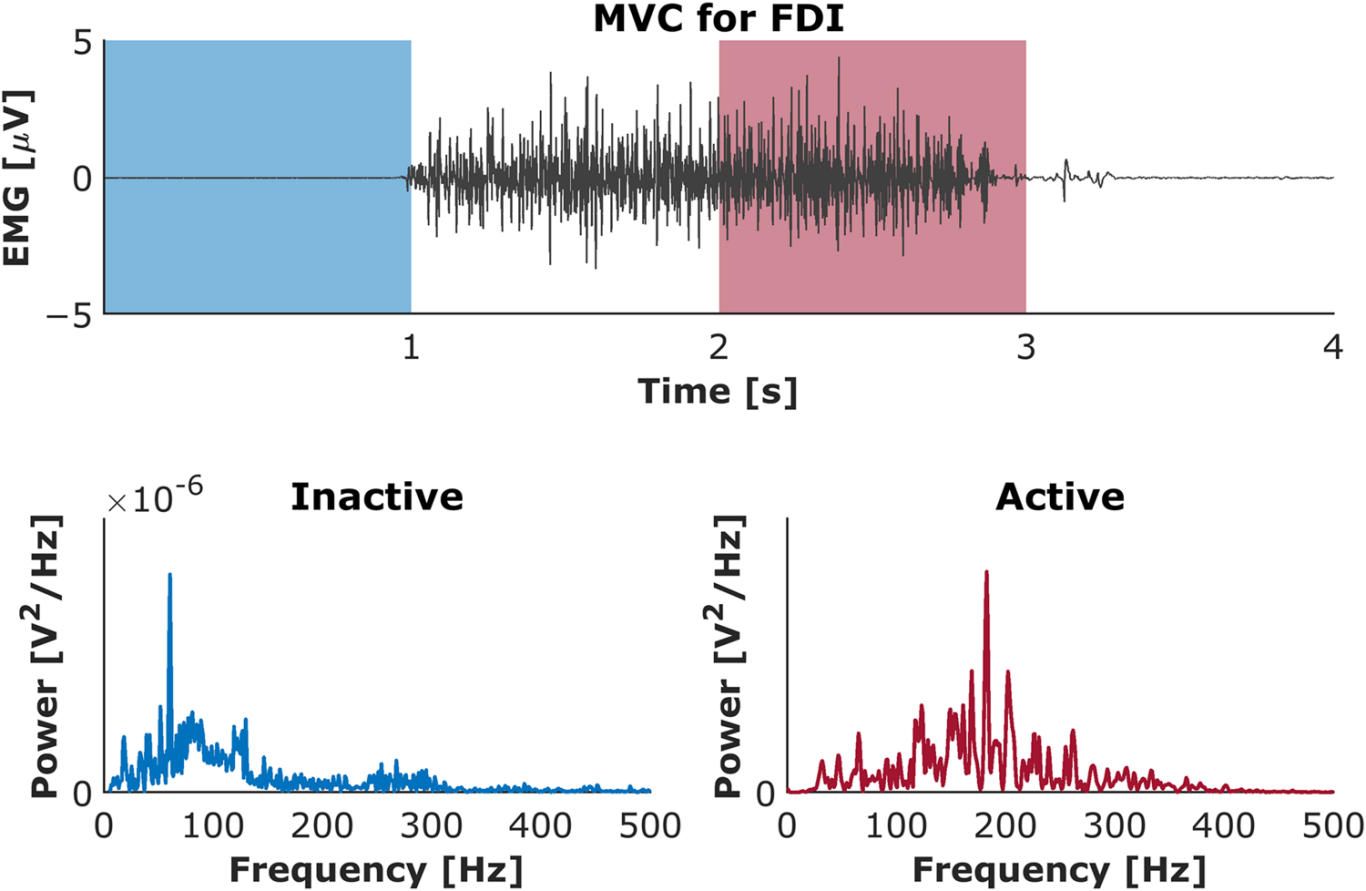
Power spectrum analysis validated all recorded EMG signals. Top panel: EMG activity recorded during maximal voluntary contraction (MVC) test for 1st dorsal interosseous muscle (FDI). Bottom panels: The distribution of the median frequency throughout the trial duration both for inactive and active muscle.

**Fig. 12:**
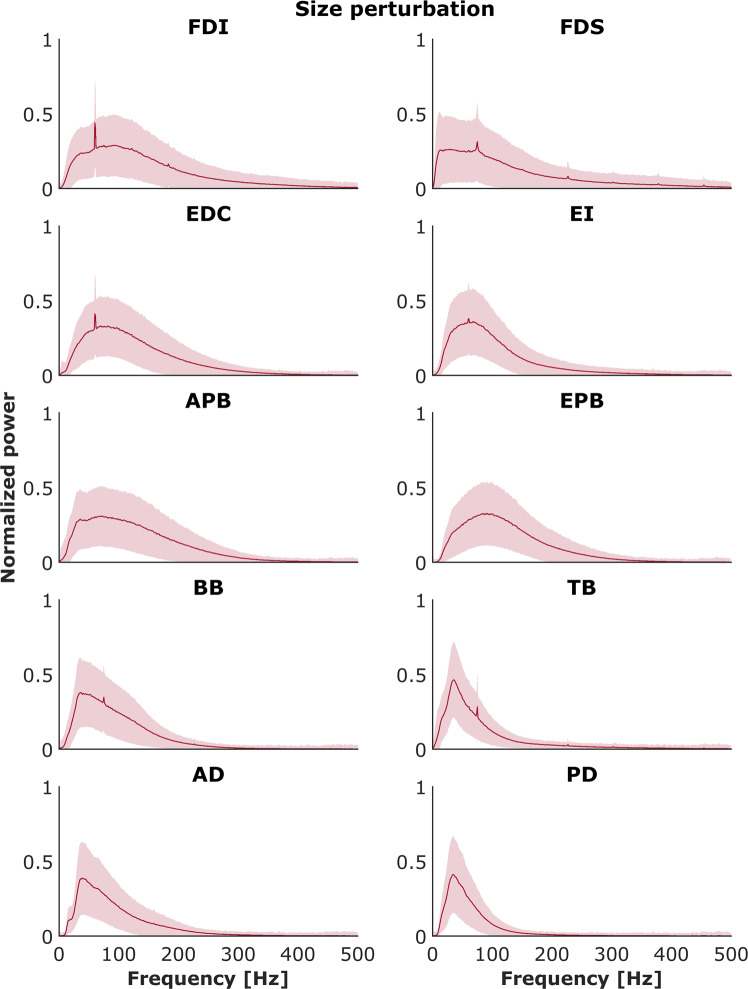
Powers spectrum densities for each muscle across all participants for the control (no perturbation) and size perturbation conditions. Shaded areas indicate ±1 SE.

**Fig. 13:**
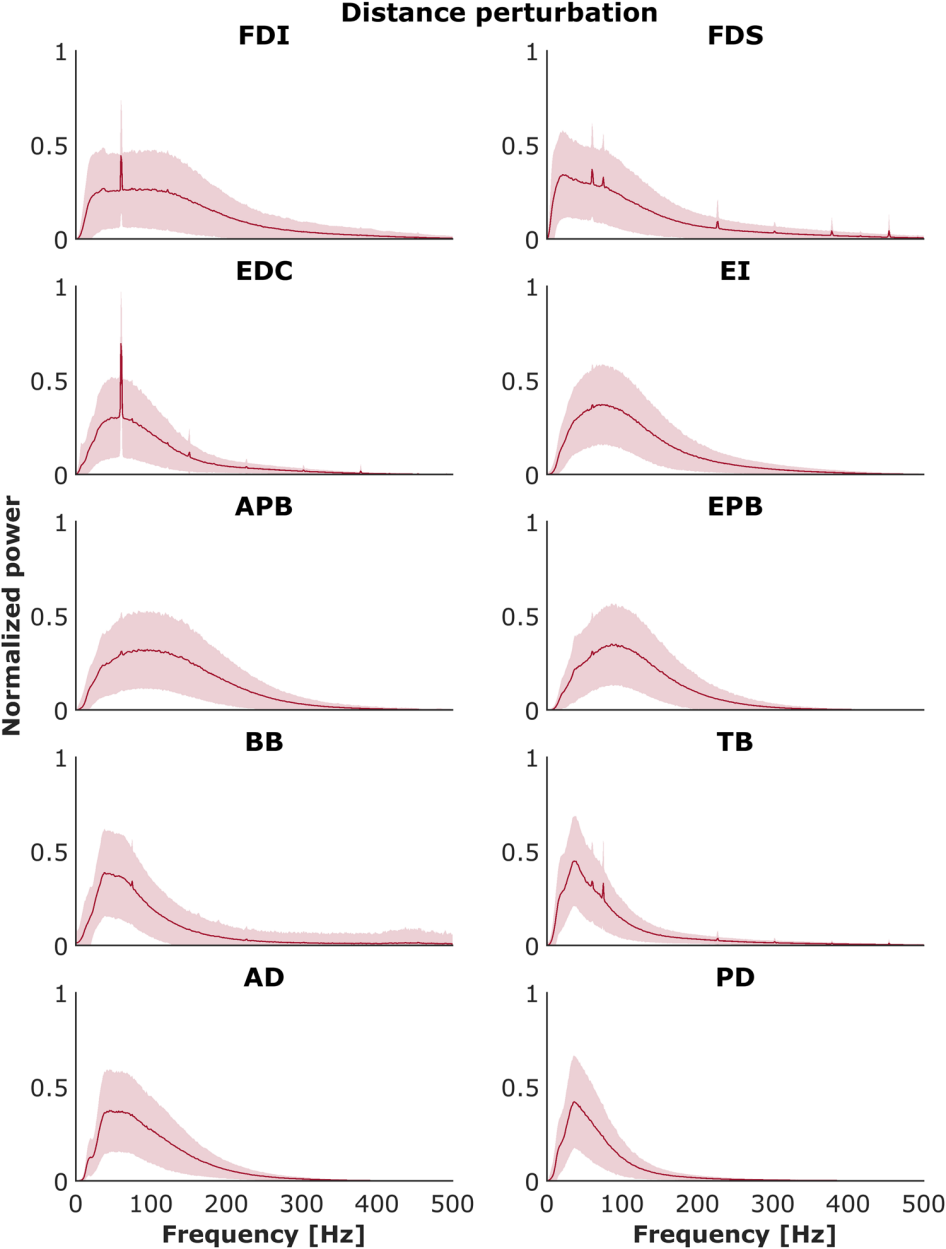
Powers spectrum densities for each muscle across all participants for the control (no perturbation) and distance perturbation conditions. Shaded areas indicate ±1 SE.

Each recorded EMG signal was validated via analysis of its spectral properties and then compared with known results from the literature. For each muscle for each trial, the power spectral density was calculated using Welch’s method with a Hann window of 1024 samples (i.e., 1024 ms) and 50% overlap. An example is presented on Fig. 11 for the EMG signal obtained during MVC test both for inactive and active muscle. Power was normalized to the maximum power on the respective trial and averaged across all trials and subjects for each muscle. Figures 12 and 13 show the mean normalized power spectral densities for EMG collected in size and distance perturbation conditions, respectively. Signal energy was primarily contained within 0–400 Hz, which is typical for EMG^51,52^. Power line noise (60 Hz) or its harmonics was observed in a minority of muscles. This artifact was a narrow band and is amenable to standard filtering procedures. In a minority of muscles, a second artifact was observed ~74 Hz. We cannot explain this artifact, but it is also a narrow band, consistent, and amenable to filtering using a band stop filter.

## Usage Notes

A major strength of the present dataset is that it provides reach-to-grasp kinematics and EMG data for a larger number of combinations of object size and distance and a large number of perturbations of object size and distance applied at three different times during the movement. Numerous examples of face validity, the degree to which our data appear to measure what was intended to be measured, are readily apparent in our data. For example, peak aperture increased with object size, peak velocity increased with object distance, and perturbed movements generally showed extended movement times compared to the analogous controls. However, the present dataset is also limited in several ways, mostly pertaining to our choice of the object type, grasp type, and kinematic recording. First, we used only one object type (a cuboid), whereas everyday reach-to-grasp movements involve diverse objects, often asymmetrical in shape. Second, the participants reached-to and grasped objects using the pincer grip, which involved only the thumb and index finger, which does not capture the full diversity of grasping movements associated with grasping the same object or objects of different size or shape^53^. Finally, we attached markers to the wrist, thumb, and index finger, which does not capture the hand’s joint angle movement. These factors might limit the range of potential uses the present dataset, but it should not preclude the modeling of reach-to-grasp movements.

## Data Availability

The code used for post-processing of the kinematic data is available at https://github.com/tuniklab/scientific-data.

## References

[1] Haggard P, Wing AM (1991). Remote responses to perturbation in human prehension. Neurosci. Lett..

[2] Haggard P, Wing A (1995). Coordinated responses following mechanical perturbation of the arm during prehension. Exp. Brain Res..

[3] Schettino LF, Adamovich SV, Tunik E (2017). Coordination of pincer grasp and transport after mechanical perturbation of the index finger. J. Neurophysiol..

[4] Desmurget M, Grafton S (2000). Forward modeling allows feedback control for fast reaching movements. Trends Cogn. Sci..

[5] Rice NJ, Tunik E, Cross ES, Grafton ST (2007). On-line grasp control is mediated by the contralateral hemisphere. Brain Res..

[6] Tunik E, Frey SH, Grafton ST (2005). Virtual lesions of the anterior intraparietal area disrupt goal-dependent on-line adjustments of grasp. Nat. Neurosci..

[7] Paulignan Y, MacKenzie C, Marteniuk R, Jeannerod M (1991). Selective perturbation of visual input during prehension movements. 1. The effects of changing object position. Exp. Brain Res..

[8] Paulignan Y, Jeannerod M, MacKenzie C, Marteniuk R (1991). Selective perturbation of visual input during prehension movements. 2. The effects of changing object size. Exp. Brain Res..

[9] Volcic R, Domini F (2016). On-line visual control of grasping movements. Exp. Brain Res..

[10] Ansuini C, Santello M, Tubaldi F, Massaccesi S, Castiello U (2007). Control of hand shaping in response to object shape perturbation. Exp. Brain Res..

[11] Popovic DB, Popovic MB, Sinkjær T (2002). Neurorehabilitation of upper extremities in humans with sensory-motor impairment. *Neuromodulation Technol*. Neural Interface.

[12] van Vliet P, Pelton TA, Hollands KL, Carey L, Wing AM (2013). Neuroscience findings on coordination of reaching to grasp an object: Implications for research. Neurorehabil. Neural Repair.

[13] Tretriluxana J (2013). Feasibility investigation of the accelerated skill acquisition program (ASAP): Insights into reach-to-grasp coordination of individuals with postacute stroke. Top. Stroke Rehabil..

[14] Popović MB (2003). Control of neural prostheses for grasping and reaching. Med. Eng. Phys..

[15] Balasubramanian K (2017). Changes in cortical network connectivity with long-term brain-machine interface exposure after chronic amputation. Nat. Commun..

[16] Mastinu E (2020). Neural feedback strategies to improve grasping coordination in neuromusculoskeletal prostheses. Sci. Rep..

[17] Laschi C (2008). A bio-inspired predictive sensory-motor coordination scheme for robot reaching and preshaping. Auton. Robots.

[18] Levine S, Pastor P, Krizhevsky A, Ibarz J, Quillen D (2017). Learning hand-eye coordination for robotic grasping with deep learning and large-scale data collection. Int. J. Rob. Res..

[19] Hoff B, Arbib MA (1993). Models of trajectory formation and temporal interaction of reach and grasp. J. Mot. Behav..

[20] Ulloa A, Bullock D (2003). A neural network simulating human reach–grasp coordination by continuous updating of vector positioning commands. Neural Networks.

[21] Rand MK, Shimansky YP, Hossain ABMI, Stelmach GE (2008). Quantitative model of transport-aperture coordination during reach-to-grasp movements. Exp. Brain Res..

[22] Bullock IM, Feix T, Dollar AM (2014). The Yale human grasping dataset: Grasp, object, and task data in household and machine shop environments. Int. J. Rob. Res..

[23] Han M, Günay SY, Schirner G, Padır T, Erdoğmuş D (2020). HANDS: A multimodal dataset for modeling toward human grasp intent inference in prosthetic hands. Intell. Serv. Robot..

[24] Garcia-Hernando, G., Yuan, S., Baek, S. & Kim, T.-K. First-person hand action benchmark with rgb-d videos and 3d hand pose annotations. in *Proceedings of the IEEE Conference on Computer Vision and Pattern Recognition* 409–419 (2018).

[25] Li, Y. & Pollard, N. S. A shape matching algorithm for synthesizing humanlike enveloping grasps. in *5th IEEE-RAS International Conference on Humanoid Robots* 442–449, 10.1109/ICHR.2005.1573607 (2005).

[26] Brahmbhatt, S., Tang, C., Twigg, C. D., Kemp, C. C. & Hays, J. ContactPose: A dataset of grasps with object contact and hand pose. in *European Conference on Computer Vision* (eds. Vedaldi, A., Bischof, H., Brox, T. & Frahm, J.-M.) 361–378, 10.1007/978-3-030-58601-0_22 (Springer International Publishing, 2020).

[27] Hu Y, Osu R, Okada M, Goodale MA, Kawato M (2005). A model of the coupling between grip aperture and hand transport during human prehension. Exp. Brain Res..

[28] Rand MK, Shimansky YP, Hossain ABMI, Stelmach GE (2010). Phase dependence of transport–aperture coordination variability reveals control strategy of reach-to-grasp movements. Exp. Brain Res..

[29] Rand MK, Shimansky YP (2013). Two-phase strategy of neural control for planar reaching movements: I. XY coordination variability and its relation to end-point variability. Exp. Brain Res..

[30] Takemura N, Fukui T, Inui T (2015). A computational model for aperture control in reach-to-grasp movement based on predictive variability. Front. Comput. Neurosci..

[31] Verheij R, Brenner E, Smeets JBJ (2012). Grasping kinematics from the perspective of the individual digits: A modelling study. PLoS One.

[32] Zhang S, Zhang Z, Zhou N (2015). A new control model for the temporal coordination of arm transport and hand preshape applying to two-dimensional space. Neurocomputing.

[33] Jarque-Bou NJ, Vergara M, Sancho-Bru JL, Gracia-Ibáñez V, Roda-Sales A (2019). A calibrated database of kinematics and EMG of the forearm and hand during activities of daily living. Sci. Data.

[34] Matran-Fernandez A, Rodríguez Martínez IJ, Poli R, Cipriani C, Citi L (2019). SEEDS, simultaneous recordings of high-density EMG and finger joint angles during multiple hand movements. Sci. Data.

[35] Gabiccini, M., Stillfried, G., Marino, H. & Bianchi, M. A data-driven kinematic model of the human hand with soft-tissue artifact compensation mechanism for grasp synergy analysis. In *2013 IEEE/RSJ International Conference on Intelligent Robots and Systems* 3738–3745, 10.1109/IROS.2013.6696890 (2013).

[36] Deimel R, Brock O (2015). A novel type of compliant and underactuated robotic hand for dexterous grasping. Int. J. Rob. Res..

[37] Katsiaris, P. T., Artemiadis, P. K. & Kyriakopoulos, K. J. Relating postural synergies to low-D muscular activations: Towards bio-inspired control of robotic hands. in *2012 IEEE 12*^*th*^*International Conference on Bioinformatics & Bioengineering (BIBE)* 245–250, 10.1109/BIBE.2012.6399682 (2012).

[38] Roda-Sales A, Vergara M, Sancho-Bru JL, Gracia-Ibáñez V, Jarque-Bou NJ (2019). Human hand kinematic data during feeding and cooking tasks. Sci. Data.

[39] Jarque-Bou NJ, Atzori M, Müller H (2020). A large calibrated database of hand movements and grasps kinematics. Sci. Data.

[40] Bianchi M, Salaris P, Bicchi A (2013). Synergy-based hand pose sensing: Reconstruction enhancement. Int. J. Rob. Res..

[41] Atzori M (2014). Electromyography data for non-invasive naturally-controlled robotic hand prostheses. Sci. Data.

[42] Luciw MD, Jarocka E, Edin BB (2014). Multi-channel EEG recordings during 3,936 grasp and lift trials with varying weight and friction. Sci. Data.

[43] Furmanek MP, Mangalam M, Yarossi M, Lockwood K, Tunik E (2021). figshare.

[44] Furmanek MP (2021). Effects of sensory feedback and collider size on reach-to-grasp coordination in haptic-free virtual reality. Front. Virtual Real..

[45] Furmanek MP, Mangalam M, Yarossi M, Lockwood K, Tunik E (2021). figshare.

[46] Furmanek MP (2019). Coordination of reach-to-grasp in physical and haptic-free virtual environments. J. Neuroeng. Rehabil..

[47] Castiello U, Bennett KMB, Stelmach GE (1993). Reach to grasp: The natural response to perturbation of object size. Exp. Brain Res..

[48] Gentilucci M, Chieffi S, Scarpa M, Castiello U (1992). Temporal coupling between transport and grasp components during prehension movements: Effects of visual perturbation. Behav. Brain Res..

[49] Chieffi S, Gentilucci M (1993). Coordination between the transport and the grasp components during prehension movements. Exp. Brain Res..

[50] Mangalam M, Yarossi M, Furmanek MP, Tunik E (2021). Control of aperture closure during reach-to-grasp movements in immersive haptic-free virtual reality. Exp. Brain Res..

[51] Clancy EA, Bertolina MV, Merletti R, Farina D (2008). Time- and frequency-domain monitoring of the myoelectric signal during a long-duration, cyclic, force-varying, fatiguing hand-grip task. J. Electromyogr. Kinesiol..

[52] Kattla S, Lowery MM (2010). Fatigue related changes in electromyographic coherence between synergistic hand muscles. Exp. Brain Res..

[53] Santello M, Flanders M, Soechting JF (1998). Postural hand synergies for tool use. J. Neurosci..

